# Key risk factors associated with fractal dimension based geographical clustering of COVID-19 data in the Flemish and Brussels region, Belgium

**DOI:** 10.3389/fpubh.2023.1249141

**Published:** 2023-11-03

**Authors:** Yessika Adelwin Natalia, Christel Faes, Thomas Neyens, Naïma Hammami, Geert Molenberghs

**Affiliations:** ^1^I-BioStat, Data Science Institute, Hasselt University, Hasselt, Belgium; ^2^I-BioStat, Leuven Biostatistics and Statistical Bioinformatics Centre, KU Leuven, Leuven, Belgium; ^3^Department of Care, Team Infection Prevention and Vaccination, Brussels, Belgium

**Keywords:** Belgium, canonical correlation analysis, COVID-19, fractal dimension, socio-demographic indicators

## Abstract

**Introduction:**

COVID-19 remains a major concern globally. Therefore, it is important to evaluate COVID-19's rapidly changing trends. The fractal dimension has been proposed as a viable method to characterize COVID-19 curves since epidemic data is often subject to considerable heterogeneity. In this study, we aim to investigate the association between various socio-demographic factors and the complexity of the COVID-19 curve as quantified through its fractal dimension.

**Methods:**

We collected population indicators data (ethnic composition, socioeconomic status, number of inhabitants, population density, the older adult population proportion, vaccination rate, satisfaction, and trust in the government) at the level of the statistical sector in Belgium. We compared these data with fractal dimension indicators of COVID-19 incidence between 1 January – 31 December 2021 using canonical correlation analysis.

**Results:**

Our results showed that these population indicators have a significant association with COVID-19 incidences, with the highest explanatory and predictive power coming from the number of inhabitants, population density, and ethnic composition.

**Conclusion:**

It is important to monitor these population indicators during a pandemic, especially when dealing with targeted interventions for a specific population.

## 1. Introduction

The transmission of coronavirus disease 2019 (COVID-19) remains a major concern globally three years after its first outbreak in Wuhan, China. The causes of COVID-19, severe acute respiratory syndrome coronavirus 2 (SARS-CoV-2) and its variants, are known to produce various signs and symptoms. The latest variants of concern, the Omicron family, caused a considerable increase in COVID-19 cases and hospitalizations in Belgium in January and February 2022 (1), with subsequent waves having lower peak values.

To keep abreast with the rapidly changing dynamics of COVID-19 transmission, it is essential to use an effective method to evaluate the evolution of this disease. Statistical models such as time series analysis or spatiotemporal modeling have been frequently used to evaluate the trends (2, 3). Mathematical modeling and systems are also used to describe and predict changes in the transmission (4). Recently, the concept of fractal dimension has emerged as a promising tool for summarizing COVID-19 data. This stems from the recognition that epidemic data tends to exhibit considerable heterogeneity, especially when observed on a smaller geographical scale, giving rise to a rather noisy dataset. Nevertheless, it is worth noting that this noise might contain valuable information. Considering the geographical scaling, we can view epidemic data as possessing a fractal nature, where the intricacies of the data can be effectively described using a fractal dimension (5). Păcurar and Necula showed that fractals were useful to assess some characteristics in an epidemic outbreak (6). A hybrid fractal theory and fuzzy logic approach has been proposed to forecast COVID-19 time series data (7). Some studies combined mathematical modeling with fractal dimensions to assess transmission and control of COVID-19 cases (8, 9). Based on this reasoning, we believe that the fractal dimension could serve as a valuable tool for assessing the local epidemic curve.

It is known that socio-demographic factors are closely associated with many infectious diseases. Many studies reported that factors such as socioeconomic status, population density, and mobility, play an important role in COVID-19 transmission (10–13). On top of these factors, race or ethnicity is also associated with varying COVID-19 incidences and outcomes. In multicultural populations, different population structures might have different COVID-19 transmission and incidence patterns. For example, compared to Caucasians, the risk for a positive COVID-19 test was increased in African and Hispanic people who live in the United States, while in the United Kingdom, the same risk is increased for African, South Asian, and Middle Eastern people (14). In Kuwait, South Asians had higher odds of mortality and intensive care admission compared to Arabs and this finding might be influenced by their socioeconomic status since the vast majority of South Asians in Kuwait were unskilled laborers living in highly populated areas (15). Baqui et al. reported higher risks of mortality among *Pardo* (people of mixed ethnic ancestries) and Black Brazilians (16).

Considering the complex nature and dynamics of socio-demographic factors, it is crucial to investigate different combinations and associations among these factors with COVID-19 indicators, not only during the pandemic but also in view of pandemic preparedness. Considerable work has been done regarding the relationship between COVID-19 risks and socio-demographic factors. Numerous studies reported the impact of multiple factors on the spread of the epidemic using diverse methodologies, including system dynamics and complex network analysis such as the susceptible-infected-recovered model and its extensions (17–19), which focus more on understanding the structure and dynamics of interconnected networks with an aim to forecast epidemic patterns such as wave durations or numbers of cases. A notable drawback of this method is the necessity to estimate the value of unknown parameters based on a limited number of observations, which poses substantial challenges, especially when dealing with complex models or frequent changes in the parameters (20). Fractal dimensions, on the other hand, seek to quantify the epidemic complexity through the collected time-series data. This complexity will then be linked to certain population characteristics. It is arguably important to assess different variable combinations to determine how these factors influence the disease indicators, especially when some factors are specific to a certain population. Thus, the novelty of this study is threefold. First, the COVID-19 incidence curves are transformed into fractal dimension related characteristics, reflected by mean, variance, and correlation functions. Second, this is done at the level of the statistical sector, a fine-grained geographical entity. Third, a rich set of explanatory factors is employed, including a detailed ethnic fingerprint of a sector. The fact that both the fractal dimension variables as well as the explanatory variables are multivariate naturally leads to canonical correlation.

In a previous study, we proposed the use of fractal dimensions combined with *k*-means clustering to classify the complexity of COVID-19 time-series data at a spatially aggregated geographical level of high resolution (21). The COVID-19 daily incidences could be explained by the estimated local fractal dimension curves and their respective mean, variance, and autocorrelation values. The unsupervised machine learning technique *k*-means clustering was used to group these indices into distinct, non-overlapping clusters. The centroid value of each cluster was subsequently compared with the mean value of each respective index. Using this approach, we were able to explore the complexity of COVID-19 time series data and characterize the epidemic behavior in a given area. In this study, we investigated the association between different socio-demographic factors and the complexity of the COVID-19 curve calculated with fractal dimension. This study contributes to the literature by combining a refined method to evaluate disease trends that can be used in small areas with routinely collected socio-demographic data to investigate probable indicators associated with variations in COVID-19 incidences.

## 2. Methods

### 2.1. Data

**Figure 1:**
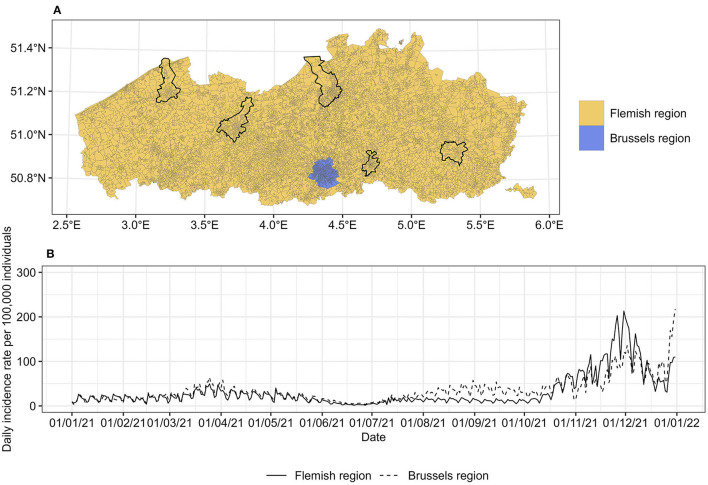
**(A)** Statistical sectors in the Flemish and Brussels region, Belgium, 2021. The capital of each province is marked with a black line. **(B)** Daily incidence rates per 100,000 individuals in each region. The map is adapted from https://statbel.fgov.be/en/open-data/statistical-sectors-2020 using R 4.2.1.

Belgium is divided into three geographical regions: Flanders, Brussels, and Wallonia. These regions consist of 300, 19, and 262 municipalities, respectively. Each municipality is further subdivided into statistical sectors. These statistical sectors, 19,794 in total, represent the smallest administrative areas in Belgium. Individual data of daily COVID-19 confirmed cases at this level were provided by the Agency for Care and Health (https://www.zorg-en-gezondheid.be/). The agency collects data from the Flemish region. Data from the Brussels region have a compatible structure and can be incorporated into the analysis. Consequently, our work focuses on these two geographical entities. In 2020, the Flemish region was divided into 9,194 statistical sectors, while there were 724 statistical sectors in the Brussels region, as shown in Figure 1A. We retrieved data from 1 January until 31 December 2021, i.e., from the start of the COVID-19 vaccination campaign in Belgium. Arguably, the vaccination rate has an impact on COVID-19 transmission, especially in the short term. The vaccination data in the Flemish region were provided by the Agency for Care and Health while the vaccination data in the Brussels region were provided by the Joint Community Commission of Brussels (https://www.ccc-ggc.brussels/nl). We calculated the vaccination rate as the percentage of fully vaccinated residents per statistical sector.

Data on socio-demographic factors at the statistical sector level were provided by StatBel, the Belgian official statistics authority (https://statbel.fgov.be/en). Key socio-demographic factors used in this study are ethnic composition, median income as a proxy for socioeconomic status, number of inhabitants, population density per km^2^, and proportion of the older adult population (50 years and older). To simplify the use of ethnic composition, we summarized this variable into the Shannon diversity index (22). A higher value of the Shannon index indicates higher ethnic diversity within a statistical sector. For the Flemish region, we also included the population proportion with high trust levels in the federal and regional government as potential factors, as well as satisfaction with the healthcare provided, based on a three-yearly survey conducted in the year 2020 at the municipality level (https://gemeente-stadsmonitor.vlaanderen.be/).

### 2.2. Statistical analysis

Each statistical sector consists of different socio-demographic factors and vaccination rates, which we further refer to as population indicators. Considering the complex nature of the COVID-19 incidence curve, we used fractal dimensions to gain a better insight into its complexity pattern. A detailed methodology of the local fractal dimension has been described in our previous work (21). We included in this paper a brief summary of the fractal dimension approach to maintain conciseness and avoid redundancies. First, we calculated the daily COVID-19 incidence rate per statistical sector based on the data provided by the Agency for Care and Health. Second, we estimated the moving fractal dimension of these daily COVID-19 incidence curves using four different methods: box-count, Hall-Wood, variogram, and madogram (23). For each method, we used three different sliding windows (of 7, 14, and 21 days) to create a local fractal dimension curve. These different sliding windows were used to assess dynamics in curves of fractals encompassing periods of varying duration. Finally, we summarized the local fractal dimension into three indicators: mean, variance, and autocorrelation value, which will be referred to as fractal dimension indicators. Considering the multiple inter-correlated fractal dimension indicators as well as various population indicators, we used canonical correlation analysis (CCA) to find a relationship between population indicators and fractal dimension indicators.

Given two sets of multiple variables *X* = *X*_1_, *X*_2_, …, *X*_*m*_ and *Y* = *Y*_1_, *Y*_2_, …, *Y*_*n*_, CCA seeks the orthogonal linear combinations of the variables within each set of indicators based on a weighted average, such that the linear combination of the *X* variables (i.e. the population indicators that include ethnic diversity index, median income, population size, population density, the proportion of older adult population, vaccination rate, trust, and satisfaction in the government), denoted as *U*, given by:


(1) $$\begin{array}{l} U=a_{1}X_{1}+a_{2}X_{2}+\cdots +a_{m}X_{m} \end{array}$$

and the combination of *Y* variables (i.e. the mean, variance, and autocorrelation value of the local fractal dimension), denoted as *V*, given by:


(2) $$\begin{array}{l} V=b_{1}Y_{1}+b_{2}Y_{2}+\cdots +b_{n}Y_{n} \end{array}$$

has a maximum correlation. *U* and *V* are the so-called canonical variates that will be used to explain the correlation both within and between sets with constraints that cov(*U*_*j*_, *U*_*k*_), cov(*V*_*j*_, *V*_*k*_), and cov(*U*_*j*_, *V*_*k*_) are equal to 0 for all *j* ≠ *k*, *j* & *k* ∈ 1, …, *i* (24). The number of canonical variates *i* is equal to the smallest set of variables so that *i* = 3 in our study. The association between *X* and *Y* variables is evaluated by means of canonical loading values, which signify the degree of correlation between these variables and their canonical variate. Higher canonical loadings serve as an indicator of a stronger association between these two variables. Additionally, the sign of a canonical loading determines the direction of their correlation. A positive loading indicates a positive contribution to the canonical correlation, thus establishing a positive association with other variables exhibiting positive loadings on the same canonical variate. This interpretation is equally applicable to negative canonical loadings, which denote a contrary orientation of association. To assess the amount of variability in the fractal dimension indicators that can be explained by the population indicators, we used the so-called redundancy analysis.

Data processing and statistical analysis were performed using R 4.2.1 available from the Comprehensive R Archive Network (CRAN) at https://CRAN.R-project.org/. CCA was performed using package candisc (25).

## 3. Results

### 3.1. Daily COVID-19 incidence

There were 536,800 cases reported between 1 January and 31 December 2021 with a known residential statistical sector. The incidence rate in both regions was relatively stable in the first half of 2021 with a slightly higher incidence in the Brussels region (Figure 1B). The incidence declined in June 2021 and then increased again in July 2021, with a large peak at the beginning of December 2021 for the Flemish region and at the end of December 2021 for the Brussels region.

### 3.2. Fractal dimension indicators

**Figure 2:**
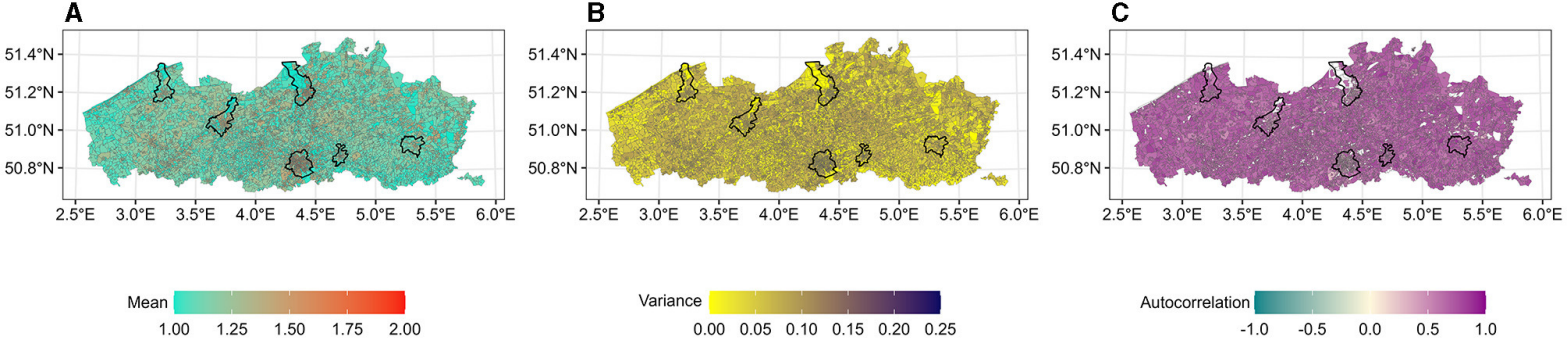
**(A–C)** Fractal dimension indicators per statistical sector calculated using box-count estimator with a 7-day sliding window. The capital of each province is marked with a black line. The white color indicates no values. The map is adapted from https://statbel.fgov.be/en/open-data/statistical-sectors-2020 using R 4.2.1.

The local fractal dimension was calculated using four estimators and three sliding windows. The fractal dimension indicators obtained from the local fractal dimension curve based on the box-count estimator with a 7-day sliding window are depicted in Figure 2. The fractal dimension indicators based on other sliding windows as well as other estimators (Hall-Wood, variogram, and madogram) are found in Supplementary Figures S1–S4. Higher mean values could be observed in many statistical sectors including larger cities such as Brussels, Antwerp, and Ghent. These higher values correspond to the higher complexity of COVID-19 incidence curves in these areas. Depending on the variance value, we observe sporadic or community transmission. In combination with higher mean values, areas with lower variance values experienced community transmission.

### 3.3. Population indicators

**Figure 3:**
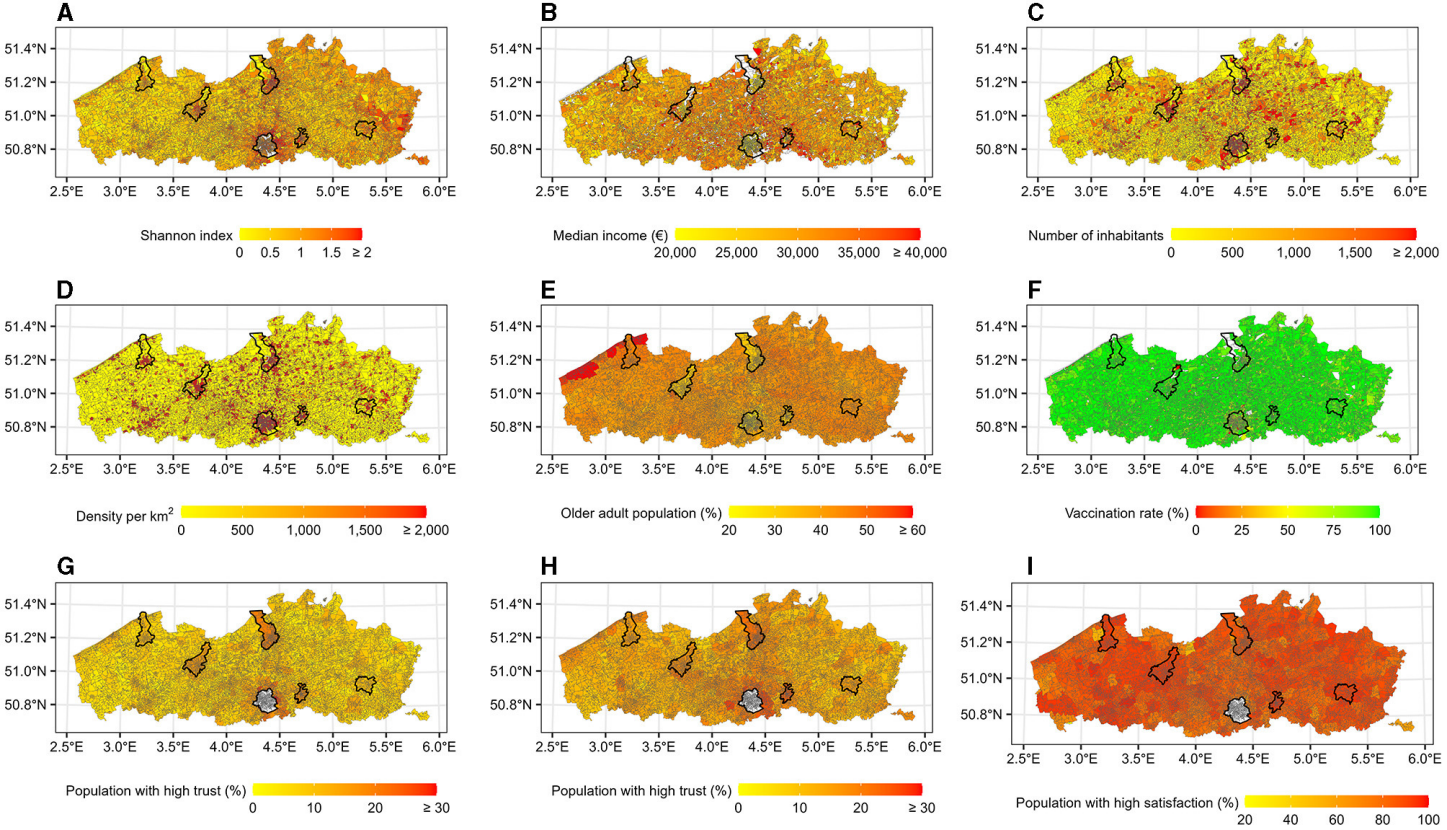
Population indicators per statistical sector. The capital of each province is marked with a black line. The white color indicates no values. **(A)** Ethnic diversity. **(B)** Socioeconomic status. **(C)** Population size. **(D)** Population density. **(E)** Older adult population (≥ 50 years). **(F)** Vaccination rate. **(G)** Trust in federal government. **(H)** Trust in regional government. **(I)** Satisfaction. The map is adapted from https://statbel.fgov.be/en/open-data/statistical-sectors-2020 using R 4.2.1.

The population in the Flemish and Brussels regions could be divided into 14 ethnic groups, based on the country or region of origin: (i) Belgium, (ii) the Netherlands, (iii) France, (iv) North and other Western European countries, (v) Southern Europe, (vi) Eastern European members of the European Union, (vii) Eastern European non-members of the European Union, (viii) Organization for Economic Co-operation and Development (OECD) countries, (ix) Maghreb countries, (x) other African countries, (xi) Asia, (xii) Turkey, (xiii) Central and South America, and (xiv) unknown origin. The Shannon diversity index ranges from 0 (no diversity) to 2.38 (very high diversity). A higher Shannon index was found in statistical sectors in larger cities as well as in the border area with the Netherlands and the prior coal mining municipalities in the eastern part of the Flemish region (Figure 3A).

The median income in each statistical sector ranges from *e*2,213 to *e*55,949, which was distributed randomly across the region (Figure 3B). In 2021, around 68% of the Belgian population lived in the Flemish and Brussels regions, with a higher number of inhabitants and population density in larger cities, particularly in the municipality of Antwerp and in the Brussels region (Figures 3C, D). Only slightly more than 39% of these inhabitants were aged 50 years and older and 43% of them resided in the Flemish region. The percentages were higher in the northeast, near the coast (Figure 3E). The vaccination rate was rather high in the Flemish region. Most statistical sectors reached a vaccination rate of 75–100% (Figure 3F), while the vaccination rate in the Brussels region was around 50–75%.

In the Flemish region, a higher proportion of people with trust in the federal, as well as the regional government, could be found in larger municipalities around the capital of each province (i.e., Antwerp, Ghent, Leuven, Hasselt, Bruges) and to some extent in the coastal areas (Figures 3G, H). Most municipalities had a high proportion of people satisfied with the healthcare provided (Figure 3I).

### 3.4. Association between population indicators and local fractal dimension curve

The mean, variance, and autocorrelation values calculated for each fractal dimension estimator based on a 7-day sliding window were grouped into fractal dimension indicators so that we have three variables in this set. Statistical sectors with missing autocorrelation values, due to no reported COVID-19 cases within the study period, were excluded. There were 9,517 statistical sectors included in this analysis. We found at least two significant correlations among three canonical variates in both regions, as shown in Tables 1, 2. For each method, the first canonical variate showed a very strong correlation and explained more than 91% of the correlation between the two sets of indicators. The canonical correlations based on longer sliding windows are presented in Supplementary Tables S1, S2.

**Table 1:** Canonical correlation between population indicators and fractal dimension indicators in the Flemish region.

**Canonical variate**	**Box-count**			**Hall-Wood**			**Variogram**			**Madogram**		
	*R*_*c*_	**Prop**	***p*** **-value**	*R*_*c*_	**Prop**	***p*** **-value**	*R*_*c*_	**Prop**	***p*** **-value**	*R*_*c*_	**Prop**	***p*** **-value**
1	0.9340	99.4514	<0.001	0.9319	99.3552	<0.001	0.9179	99.2996	<0.001	0.9134	99.3681	<0.001
2	0.1606	0.3852	<0.001	0.1625	0.4084	<0.001	0.1716	0.5633	<0.001	0.1654	0.5562	<0.001
3	0.1054	0.1634	<0.001	0.1243	0.2364	<0.001	0.0856	0.0074	<0.001	0.0618	0.0038	<0.001

The local fractal dimension was calculated using a 7-day sliding window.

*R*_*c*_, canonical correlation value; prop, proportion of the correlation explained by this canonical variate.

**Table 2:** Canonical correlation between population indicators and fractal dimension indicators in the Brussels region.

**Canonical variate**	**Box-count**			**Hall-Wood**			**Variogram**			**Madogram**		
	*R*_*c*_	**Prop**	***p*** **-value**	*R*_*c*_	**Prop**	***p*** **-value**	*R*_*c*_	**Prop**	***p*** **-value**	*R*_*c*_	**Prop**	***p*** **-value**
1	0.8949	99.3100	<0.001	0.9133	99.1930	<0.001	0.8526	98.5700	<0.001	0.8583	98.7406	<0.001
2	0.1633	0.6770	0.0430	0.1714	0.0303	0.0022	0.1863	1.3317	0.0042	0.1601	10.2400	0.0079
3	0.0269	0.0179	0.9745	0.1026	0.2100	0.1273	0.0509	0.0960	0.7811	0.0964	0.3309	0.1766

The local fractal dimension was calculated using a 7-day sliding window.

*R*_*c*_, canonical correlation value; prop, proportion of the correlation explained by this canonical variate.

The canonical loading between each set of indicators and their canonical variates are shown in Table 3 (results based on longer sliding windows can be found in Supplementary Table S3). We confined our attention to the first canonical variate. Some population indicators have more explanatory power than others, mainly when the absolute value of the canonical loading is above 0.5 such as population size and population density. The first canonical variate was represented strongly by mean, variance, and autocorrelation values for fractal dimension indicators. In the Flemish region, the canonical loadings of population size, population density, Shannon index, satisfaction, and trust in the government have a sign consistent with that of the mean. A consistent sign indicates changes in the same direction, i.e., a higher value of these population indicators would result in higher mean and variance values. In contrast, older adult proportion, median income, and vaccination rate have a sign opposite to that of mean and variance, indicating lower mean and variance values when the observed population indicators are higher. The Brussels region showed similar trends except for predominantly positive canonical loading of the fractal dimension variance.

**Table 3:** Canonical loading between each set of indicators and their first canonical variate.

**Variable**	**Flemish region**				**Brussels region**			
	**Box-count**	**Hall-Wood**	**Variogram**	**Madogram**	**Box-count**	**Hall-Wood**	**Variogram**	**Madogram**
Population size	**-0.9907**	**-0.9914**	**-0.9895**	**-0.9898**	**-0.9898**	**-0.9950**	**-0.9855**	**-0.9850**
Population density	**-0.6876**	**-0.6831**	**-0.6909**	**-0.6894**	**-0.6105**	**-0.5879**	**-0.6236**	**-0.6180**
Shannon index	**-0.3671**	**-0.3641**	**-0.3755**	**-0.3755**	**-0.2570**	**-0.2177**	**-0.2650**	**-0.2419**
Older adult population	0.2832	0.2839	0.2826	0.2821	0.3257	0.3070	0.3121	0.3021
Median income	0.2945	0.2883	0.3011	0.2986	0.3288	0.3209	0.3290	0.3289
Vaccination rate	0.2118	0.2098	0.2098	0.2087	0.2070	0.2276	0.1748	0.2020
Satisfaction	**-0.0695**	**-0.0658**	**-0.0737**	**-0.0731**				
Trust in the federal government	**-0.3323**	**-0.3269**	**-0.3414**	**-0.3396**				
Trust in the regional government	**-0.2610**	**-0.2549**	**-0.2751**	**-0.2741**				
Mean FD	**-0.9392**	**-0.9458**	**-0.9169**	**-0.9241**	**-0.8099**	**-0.7902**	**-0.5305**	**-0.6191**
Variance FD	**-0.6360**	**-0.7292**	**-0.1455**	**-0.4270**	0.1411	0.0413	0.9731	0.7258
ACF FD	0.7086	0.1036	0.8280	0.6803	0.9179	0.2693	0.7608	0.5937

Population and fractal dimension indicators with consistent negative signs are marked in bold.

ACF, autocorrelation value; FD, fractal dimension.

Depending on the method used, the so-called redundancy analysis showed that 26–53% of the total variability in fractal dimension indicators is explained by the changes in all population indicators in the Flemish region, while the analysis in the Brussels region ranged from 13 to 45%. The box-count estimator showed the highest total variability in fractal dimension indicators which can be explained by changes in population indicators for both regions.

## 4. Discussion

Based on the canonical correlation analysis results, we found a strong association between population indicators and fractal dimension indicators of the COVID-19 incidence curve. This suggests an effect of socio-demographic factors on COVID-19 incidence.

Our results showed that population size, population density, and the Shannon diversity index have the strongest influence on the complexity of the COVID-19 incidence curve. Higher values of these population indicators were associated with higher complexity of the COVID-19 incidence curve, which further indicates a possible community transmission. We also found associations between a lower proportion of the older adult population, income, and vaccination rate with the increased complexity of the COVID-19 incidence curve.

Similar findings were reported in other studies. A study in the southern part of Brazil showed a strong positive correlation between population size and the number of COVID-19 cases and deaths (26). Studies in the US and China reported higher numbers of COVID-19 cases and deaths in urban or densely populated areas (10, 27). Oh et al. found a significant influence of many socio-demographic factors on COVID-19 incidences, such as racial and ethnic composition, age, income, household size, and population density (28). In contrast to these findings, a study in the Latin American and Caribbean countries showed that countries with higher numbers of inhabitants per square kilometer had lower death rates (29). Differences in these findings might be attributed to inequalities in the population. Molallo *et al*. reported that a higher income inequality, defined as the ratio of household income at the 80^th^ percentile to income at the 20^th^, is an influential factor in explaining the increase in COVID-19 incidence, particularly in the tri-state area in the US (30). We could see a similar trend in Table 3 where the lower median income is associated with higher mean and variance values of the fractal dimension indicators. Moreover, there was also inequity in healthcare associated with access and quality of healthcare (31).

Another socio-demographic factor that should be considered is social contact patterns, which vary by age, gender, and location (32). In Belgium, a shift in COVID-19 transmission to the younger age group was reported in the Fall of 2020 (33). This is again reflected in Table 3, where a lower proportion of the older adult population is associated with higher mean and variance values. Many studies reported similar trends, even though severe morbidity and high mortality rates remained in the older age group (34–36). Increased COVID-19 cases in the younger age group could enhance community transmissions since people in this age group have complex social contact patterns. Such complex patterns can also be seen in a population with high diversity. People from foreign origins are perceived to have higher social contacts compared to local residents (37, 38). However, we should also consider different types of contacts made within the population. For example, in the U.S., Dorèlien et al. reported that Hispanic people have the highest number of household contacts while Non-Hispanic Black people have the lowest number and shortest duration of household member contacts compared to other ethnic groups at nearly all age groups. However, they also tend to have a higher proportion of jobs with the highest level of physical proximity, which increases the risks of contracting COVID-19 (39).

Another contrast in the social contact patterns could be observed based on the canonical loading of the Shannon index on the fractal dimension variance. While a higher Shannon index led to a higher mean fractal dimension, the variance would be higher only in the Flemish region. We expected that people in homogeneous areas (Shannon index closer to zero) would have more contact in their local community while people in heterogeneous areas would have more contact outside their community. Nevertheless, in cases where homogeneous areas are predominantly populated by people from foreign origins, there was a possibility that they also made contact with their home country. For example, when the travel restrictions were lifted, many people traveled long distances which eventually contributed to the increase in local COVID-19 cases, as reported in Ukraine and Taiwan in the Summer of 2021 (40, 41).

We expected that the vaccination rate would be a strong explanatory variable of the complexity of the COVID-19 incidence curve. However, we found a relatively low explanatory power of the vaccination rate on the first canonical variate, even though the vaccination rate was rather high. Vaccination showed a favorable effect on alleviating the burden of COVID-19, for example by reducing COVID-19 infection, severity, hospitalization, and mortality in the first period of the pandemic (42, 43). However, the protection wanes over time, and at some point the vaccination aids in reducing the severity or mortality but less on the transmission, especially with a new variant of concern (44). Moreover, a social contact study showed an increasing daily mean number of contacts following summer vacation in 2021 in Belgium (45). Hence, it is possible to observe many COVID-19 cases in areas with high vaccination coverage during the study period. On top of this, there were still some areas where people were very hesitant to get vaccinated. Faes et al. showed that areas in the Flemish region with a more diverse population or lower socioeconomic status have a lower vaccination coverage, even though the vaccinations were given freely (46). This created further an imbalance in the overall vaccination rate as observed in several parts of the Flemish region and particularly in the Brussels region.

We also found low explanatory power of satisfaction and trust in the government in the Flemish region, with contradictory interpretations. There was a correlation between a higher proportion of satisfaction and trust with higher mean and variance values, which indicate a higher complexity of COVID-19 incidences. We expected that higher satisfaction and trust in the government would lead to an increased willingness to follow government policies, especially in a time of crisis. Some studies reported that public satisfaction and trust in a government played an important role in the decision to be vaccinated (47, 48). A possible explanation for our finding is that higher satisfaction and trust were found in larger municipalities where we observed a higher complexity of the COVID-19 incidence curve. On the other hand, there is also a possibility of COVID-19 under-reporting in areas with lower satisfaction and trust in the government. Moreover, the survey was conducted with an online self-administered questionnaire among representative residents aged 17–85, thus it is possible that selection bias occurs. Despite this contradictory finding, we believe in the importance of building good government-public relationships to improve the pandemic situation.

As a sensitivity analysis, we compared our findings at the level of the Flemish region to the provincial level. The results and interpretation at the provincial level were similar to the regional level, i.e., there was good explanatory power of population size, population density, and the Shannon diversity index. We found, however, a low canonical loading of the vaccination rate in the province of West Flanders. This could be explained by the relatively high and similar vaccination rates among the statistical sectors in this province in combination with high COVID-19 cases.

One of the strengths of our study lies in the fine-scale administrative unit used. We also used methods that are effective for areas with different scales, especially when we need to analyze various parameters simultaneously. Different interpretations in the results, however, should be expected when comparing different geographical units (e.g., province or municipality) due to the difference in the population as well as fractal dimension indicators within and between these areas. However, it is still possible to translate these findings into a tool that can be used by local authorities to tailor their interventions.

Despite these strengths, we also noted some limitations. First, we used the vaccination rate at the end of our study period (31 December 2021). We compared our findings to a divided study period (1 January–30 June 2021 and 1 July–31 December 2021) considering the changes in the vaccination campaign strategy. Unfortunately, we did not observe considerable differences in the explanatory power of the population indicators, including the vaccination rate. It might be interesting to also capture the changes in vaccination rate over time and use these changes as an explanatory variable. Second, there were some discrepancies in the vaccination data. Some people could be vaccinated outside of their permanent address or the number of vaccinated people registered to certain statistical sectors is higher than the registered population count, particularly when the population count is zero. For practical reasons, we removed statistical sectors with zero population from our canonical correlation analysis.

While the use of canonical correlation analysis is relatively common in the field of infectious diseases or public health, the concept of fractal dimension remains relatively unexplored within this domain. Even though the general idea of fractal dimensions might be intuitive, understanding its mathematical intricacies can become more challenging. Hence, persuading individuals to embrace the regular utilization of fractal dimensions, particularly as we intend to integrate this concept into a surveillance system, could pose a challenge. In addition to this, numerous studies lean more toward utilizing scan statistics or model-based analysis methods to assess clusters or risk factors based on anomalies in space and/or time [e.g. (49–51)]. The goal of our proposed method is to capture the complexity of a multivariate set of outcomes through time and subsequently categorize these outcomes based on their respective complexity characteristics. Therefore, we deliberately avoid imposing a spatial mechanism as the underlying data-generation process. This approach allows us to gain insights into data complexity regardless of their geographical location. Through this study, we want to underscore the potential of fractal dimension-based analysis to compare data on disease patterns and/or risk factors across diverse geographic locations, since we found (dis-)similarities with other studies.

In conclusion, our study has demonstrated a significant association between COVID-19 incidences and a range of factors at the statistical sector level, including ethnic composition, socioeconomic status, number of inhabitants, population density, the older adult population proportion, vaccination rate, satisfaction, and trust in government. To gain better control of the pandemic, it is highly relevant to monitor these population indicators. Targeted interventions such as community-oriented campaigns promoting preventive measures across various languages or the adoption of diverse vaccination strategies utilizing local institutions such as schools and workplaces, could be tailored based on the influential population indicators. It should be noted that the dynamics of an epidemic are influenced by a multitude of contributing variables. Through this study, we have demonstrated the feasibility of employing fractal dimension analysis combined with routinely collected data to interpret the epidemic patterns and identify the underlying characteristics that exhibit a robust association with epidemic propagation. It is certainly possible to incorporate additional variables as deemed necessary, such as replication rate or recovery rate, which enhances the versatility and practical utility of this approach.

## Data availability statement

The datasets presented in this article are not readily available because the daily COVID-19 cases and vaccination that support the findings of this study are available from the Agency for Care and Health as well as the Joint Community Commission of Brussels but restrictions apply to the availability of these data, which were used under license for the current study, and so are not publicly available. Other data used in this study are publicly available. Requests to access the datasets should be directed to YN, yessikaadelwin.natalia@uhasselt.be.

## Author contributions

YN participated in the design, data acquisition, analysis, visualization, and writing the original draft. GM was involved in the study conception, design, data acquisition, editing, and review of the manuscript. CF, TN, and NH were involved in the interpretation of the results and critical revision of the manuscript. All authors approved the final version of the manuscript.
