# Supplementary figures and images for: Key risk factors associated with fractal dimension based geographical clustering of COVID-19 data in the Flemish and Brussels region, Belgium
Source: Front Public Health. 2023 Nov 3;11:1249141. doi: 10.3389/fpubh.2023.1249141 (PMC10654974; doi:10.3389/fpubh.2023.1249141)

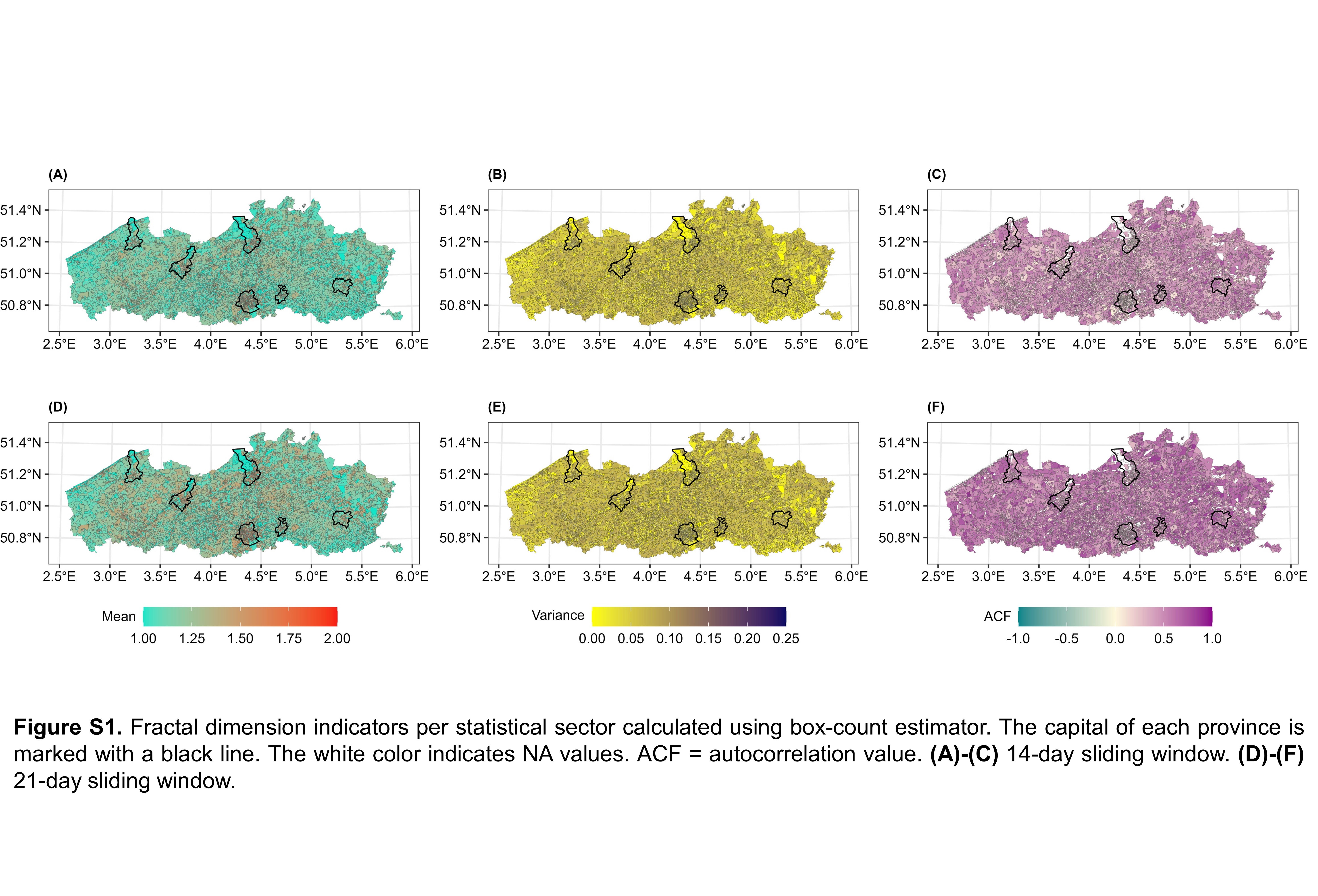

Supplement: Supplementary file 1 [file Image_1.JPEG]

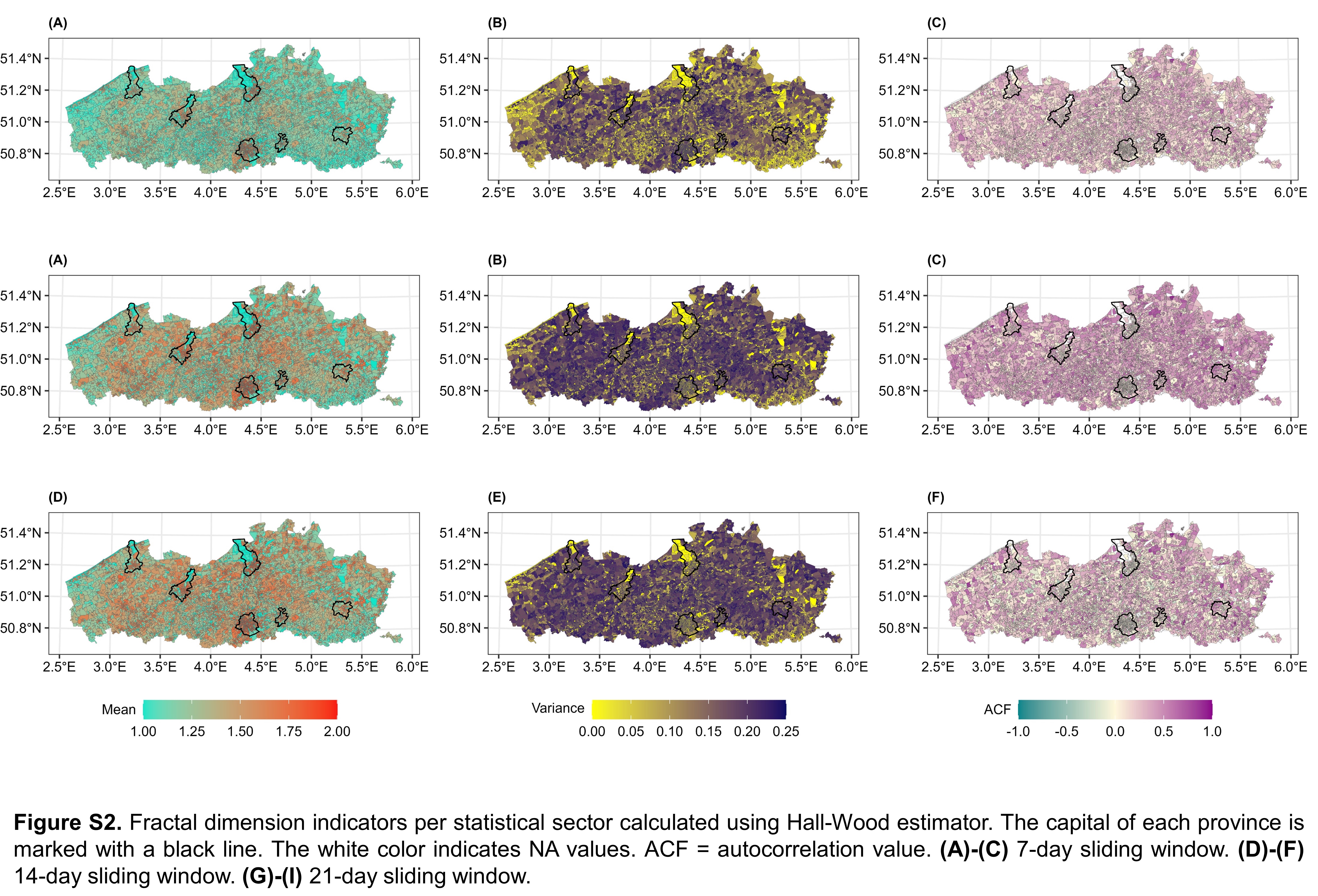

Supplement: Supplementary file 2 [file Image_2.JPEG]

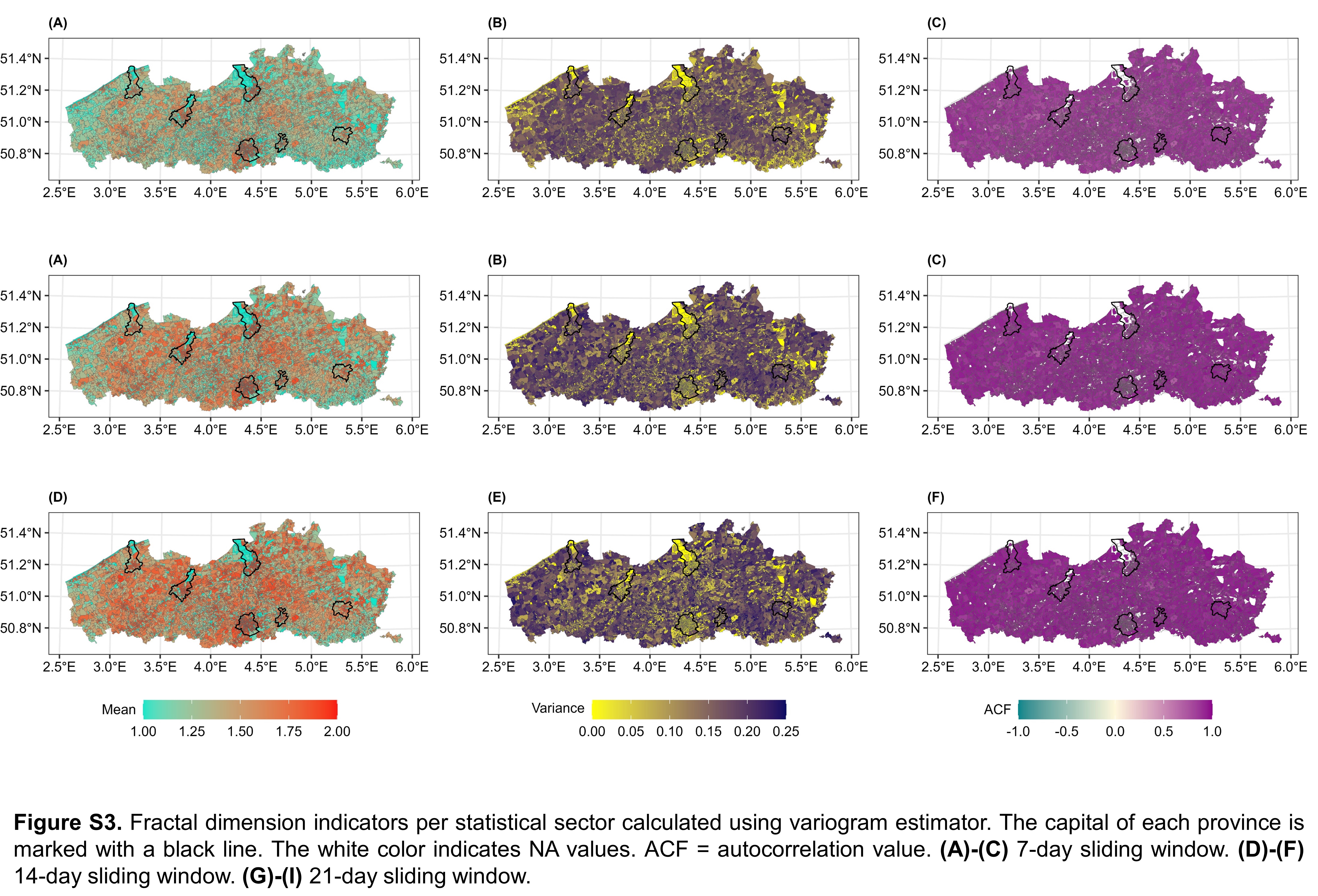

Supplement: Supplementary file 3 [file Image_3.JPEG]

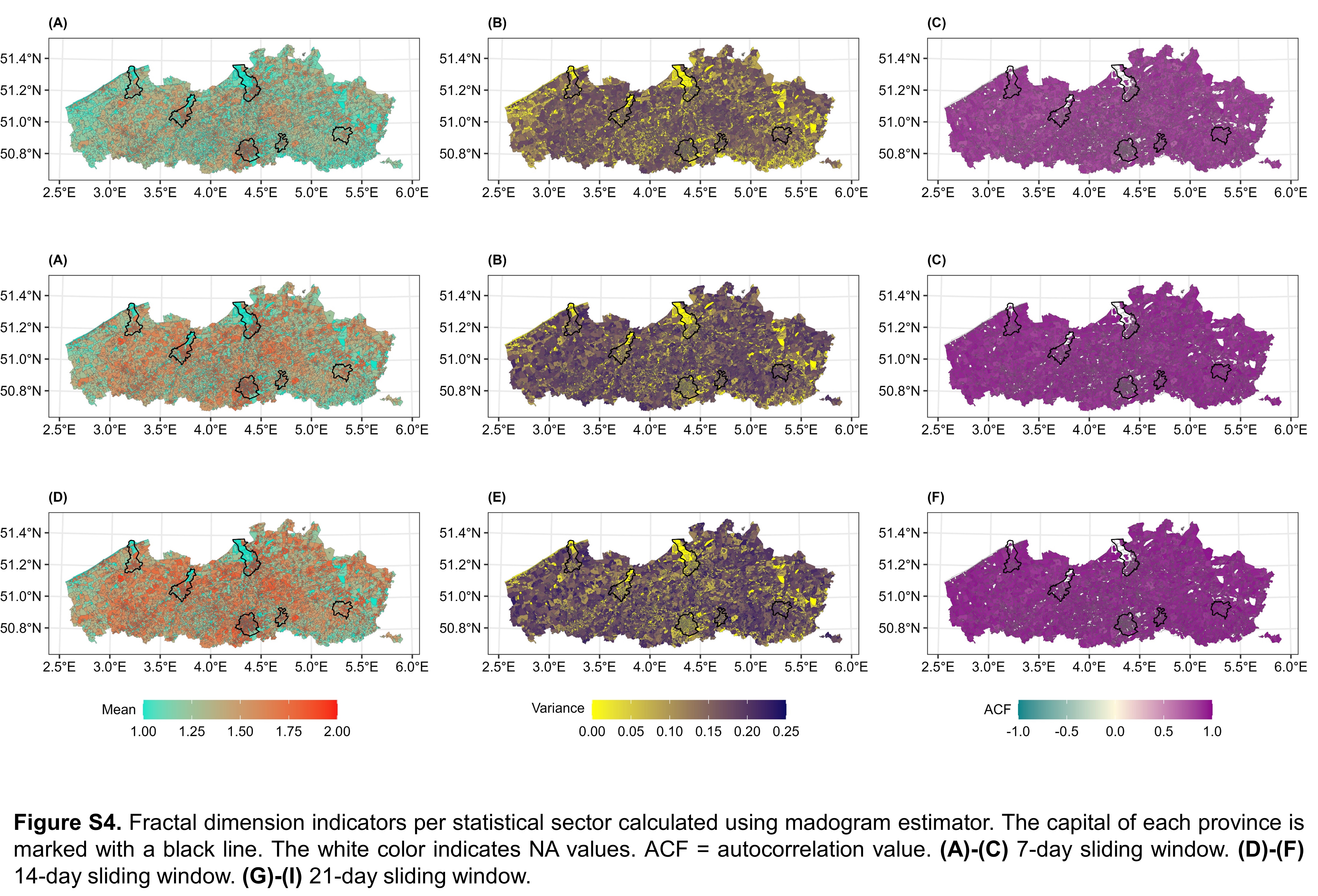

Supplement: Supplementary file 4 [file Image_4.JPEG]
