# Supplementary material for: Key risk factors associated with fractal dimension based geographical clustering of COVID-19 data in the Flemish and Brussels region, Belgium
Source: Front Public Health. 2023 Nov 3;11:1249141. doi: 10.3389/fpubh.2023.1249141 (PMC10654974; doi:10.3389/fpubh.2023.1249141)
Supplement: Supplementary file 5 [file Data_Sheet_1.PDF]

Supplementary Material

1 SUPPLEMENTARY TABLES

Table S1. Canonical correlation between population indicators and fractal dimension indicators in the Flemish region.

Canonical variate	Box-count			Hall-Wood			Variogram			Madogram		
	R_c	Prop	p-value	R_c	Prop	p-value	R_c	Prop	p-value	R_c	Prop	p-value
Sliding window = 14 days												
1	0.9187	99.3060	< 0.001	0.9092	99.1913	< 0.001	0.8737	99.1423	< 0.001	0.8702	99.2131	< 0.001
2	0.1468	0.4042	< 0.001	0.1648	0.5805	< 0.001	0.1503	0.7098	< 0.001	0.1474	0.7066	< 0.001
3	0.1247	0.2899	< 0.001	0.1042	0.2282	< 0.001	0.0692	0.1478	< 0.001	0.0502	0.0804	0.0023
Sliding window = 21 days												
1	0.9107	99.1130	< 0.001	0.8876	99.1439	< 0.001	0.8330	99.0777	< 0.001	0.8317	99.1406	< 0.001
2	0.1687	0.5973	< 0.001	0.1492	0.6082	< 0.001	0.1356	0.8184	< 0.001	0.1331	0.7972	< 0.001
3	0.1184	0.2897	< 0.001	0.0959	0.2479	< 0.001	0.0487	0.1039	0.0038	0.0375	0.0623	0.0871

R_c = canonical correlation value; prop = proportion of the correlation explained by this canonical variate.

Table S2. Canonical correlation between population indicators and fractal dimension indicators in the Brussels region.

Canonical variate	Box-count			Hall-Wood			Variogram			Madogram		
	R_c	Prop	p-value	R_c	Prop	p-value	R_c	Prop	p-value	R_c	Prop	p-value
Sliding window = 14 days												
1	0.8657	98.4900	< 0.001	0.8592	98.4647	< 0.001	0.7561	96.1358	< 0.001	0.7958	97.2896	< 0.001
2	0.1668	0.9425	0.0007	0.1814	1.1877	0.0011	0.2181	3.5967	0.0001	0.1906	2.1241	0.0004
3	0.1299	0.5656	0.0212	0.0993	0.3476	0.1521	0.0608	0.2676	0.6428	0.1015	0.5863	0.1353
Sliding window = 21 days												
1	0.8418	98.0193	< 0.001	0.8170	97.3013	< 0.001	0.6677	91.3849	< 0.001	0.7105	94.4150	< 0.001
2	0.1885	1.4850	0.0003	0.2012	2.0444	< 0.001	0.2554	7.9306	< 0.001	0.2141	4.4510	< 0.001
3	0.1102	0.4957	0.0819	0.1154	0.6543	0.0592	0.0774	0.6846	0.3970	0.1100	1.1340	0.0833

R_c = canonical correlation value; prop = proportion of the correlation explained by this canonical variate.

Table S3. Canonical loading between each set of indicators and their first canonical variate. Population and fractal dimension indicators with consistent negative signs are marked in bold.

Variable	Flemish region				Brussels region			
	Box-count	Hall-Wood	Variogram	Madogram	Box-count	Hall-Wood	Variogram	Madogram
Sliding window = 14 days								
Population size	**-0.9915**	**-0.9906**	**-0.9902**	**-0.9902**	**-0.9892**	**-0.9878**	**-0.9643**	**-0.9801**
Population density	**-0.6854**	**-0.6860**	**-0.6851**	**-0.6854**	**-0.6123**	**-0.6172**	**-0.6517**	**-0.6412**
Shannon index	**-0.3614**	**-0.3722**	**-0.3748**	**-0.3749**	**-0.2498**	**-0.2550**	**-0.2855**	**-0.2497**
Elderly population	0.2786	0.2785	0.2689	0.2704	0.3010	0.3140	0.2726	0.2816
Median income	0.2969	0.2982	0.3028	0.3011	0.3051	0.3324	0.3469	0.3497
Vaccination rate	0.2097	0.2079	0.2027	0.2022	0.1737	0.1863	0.1040	0.1648
Satisfaction	**-0.0753**	**-0.0738**	**-0.0876**	**-0.0842**				
Trust in the federal government	**-0.3256**	**-0.3357**	**-0.3369**	**-0.3375**				
Trust in the regional government	**-0.2565**	**-0.2700**	**-0.2801**	**-0.2800**				
Mean FD	**-0.9045**	**-0.9039**	**-0.8803**	**-0.8838**	**-0.5702**	**-0.4065**	**-0.0532**	**-0.1164**
Variance FD	**-0.4295**	**-0.1349**	0.5377	0.3228	0.7010	0.9924	0.8466	0.8839
ACF FD	0.6172	0.5621	0.8316	0.5956	0.4686	0.3604	0.3965	0.1207
Sliding window = 21 days								
Population size	**-0.9911**	**-0.9908**	**-0.9908**	**-0.9907**	**-0.9835**	**-0.9836**	**-0.9434**	**-0.9670**
Population density	**-0.6849**	**-0.6850**	**-0.6803**	**-0.6822**	**-0.6277**	**-0.6298**	**-0.6739**	**-0.6656**
Shannon index	**-0.3689**	**-0.3722**	**-0.3707**	**-0.3718**	**-0.2672**	**-0.2545**	**-0.2868**	**-0.2589**
Elderly population	0.2764	0.2736	0.2543	0.2576	0.3275	0.3176	0.2368	0.2560
Median income	0.3013	0.2988	0.2999	0.2986	0.3453	0.3363	0.3417	0.3441
Vaccination rate	0.2065	0.2049	0.1966	0.1969	0.1794	0.1687	0.0593	0.1283
Satisfaction	**-0.0746**	**-0.0785**	**-0.0100**	**-0.0947**				
Trust in the federal government	**-0.3293**	**-0.3345**	**-0.3274**	**-0.3310**				
Trust in the regional government	**-0.2622**	**-0.2722**	**-0.2786**	**-0.2805**				
Mean FD	**-0.9155**	**-0.8883**	**-0.8568**	**-0.8565**	**-0.5473**	**-0.3067**	0.0244	**-0.0726**
Variance FD	**-0.2268**	0.0741	0.7990	0.6940	0.8589	0.9945	0.7569	0.8249
ACF FD	0.4022	0.4250	0.8384	0.5705	0.2993	0.1441	0.3953	0.1844

ACF = autocorrelation value; FD = fractal dimension.
